# Safer esports for players, spectators, and bettors: Issues, challenges, and policy recommendations

**DOI:** 10.1556/2006.2023.00012

**Published:** 2023-03-24

**Authors:** Andrea Czakó, Orsolya Király, Patrik Koncz, Shu M. Yu, Harshdeep S. Mangat, Judith A Glynn, Pedro Romero, Mark D Griffiths, Hans-Jürgen Rumpf, Zsolt Demetrovics

**Affiliations:** 1 Centre of Excellence in Responsible Gaming, University of Gibraltar, Gibraltar, Gibraltar; 2 Institute of Psychology, ELTE Eötvös Loránd University, Budapest, Hungary; 3 Doctoral School of Psychology, ELTE Eötvös Loránd University, Budapest, Hungary; 4 International Gaming Research Unit, Psychology Department, Nottingham Trent University, Nottingham, United Kingdom; 5 Department of Psychiatry and Psychotherapy, University of Lübeck, Lübeck, Germany

**Keywords:** esport, videogame playing, gambling, betting, problematic gaming, policy

## Abstract

The present paper provides an overview of the possible risks, harms, and challenges that might arise with the development of the esports field and pose a threat to professional esports players, spectators, bettors and videogame players, including underage players. These include physical and mental health issues, gambling and gambling-like elements associated with videogames and esports, the challenges arising from pursuing a career in esports, the unique difficulties women face, and a need for supporting professional esports players. It briefly discusses possible responses and suggestions regarding how to address and mitigate these negative consequences. It emphasizes the need for cooperation and collaboration between various stakeholders: researchers, policymakers, regulators, the gaming industry, esports organizations, healthcare and treatment providers, educational institutes and the need for further evidence-based information.

Videogame playing is one of the most popular current leisure time activities. Its evolution includes the professionalization of this activity in the form of competitive gaming and now known as electronic sports (esports) (Faust, Meyer, & Griffiths, 2013). Similar to traditional sports, esports has matured into an independent branch of sport with professional teams, esports clubs, esports coaches and psychologists, official sponsorships, and broadcast tournaments with a large pool of prizes and a continuously growing audience (Taylor, 2012). The esports industry has steadily increased in revenue, as well as an increase in the number of tournaments, players, viewers, and other stakeholders (eMarketer, 2022).

A career as an esports player has become a viable option for youth due to the growing number of esports tournaments, organizations, schools, and university programs and scholarships, as well as sponsorships (Funk, Pizzo, & Baker, 2018). On a personal level, playing videogames competitively has several advantages. In addition to the potential opportunity to earn money and prestige, players can develop their skills (Bediou et al., 2018) and become a part of a larger gaming community. Simultaneously, on a community level, esports provides a new form of entertainment via online and offline tournaments, content streamed by esports celebrities, and esports betting (Greer, Rockloff, Browne, Hing, & King, 2019; Griffiths, 2017; Taylor, 2012). Furthermore, the growing esports industry stimulates the increase of several related areas and services, providing job opportunities for team managers, coaches, game analysts, marketing specialists, and broadcasters (Funk et al., 2018).

Alongside the positive aspects of esports, there are several possible threats and challenges, which should be considered. The main concerns comprise negative consequences of the sedentary lifestyle and decreased levels of physical activity, sleep disturbances, the risk of development of problematic gaming or gaming disorder, exposure to age-inappropriate or otherwise potentially harmful content (e.g., swearing, violence, sexually-explicit content, unrealistic body images, promotion of hateful or harmful behaviors, and drug use) (Bányai, Griffiths, Demetrovics, & Király, 2019; Barlett & Harris, 2008; Chan et al., 2022), and esports-related gambling (Macey & Hamari, 2019), all of which may affect both esports players and spectators. In addition, the esports career path requires huge sacrifices usually at a young age, which may negatively affect educational progression, and social relationships, as well as the risk of early burnout (Salo, 2021).

It is of paramount importance to acknowledge such risks and threats in the process of esports development and to focus on the support and protection of both professional esports players, who are often minors, and those who engage in activities related to esports. These could include spectators, young videogame players and their families, and those who gamble on esports.

## Potential risks, needs, and possible responses related to esports

The remainder of this paper provides an overview of the potential risks, threats, and challenges that might arise or increase with the development of the esports field, together with some suggestions on how to address or mitigate these negative consequences. Although most of these are outlined in relation to professional esports players, it needs to be emphasized that the majority of these issues also pose a threat to non-professional videogamers, especially minors and young players. The areas and suggestions are also summarised in Table 1.

**Table 1.** Safer esports related issues and suggestions for possible responses

Issues	Suggestions
Physical health	Providing reliable information, education and counselling on the possible negative consequences of extended play on players' bodies and lifestyle, the importance of a balanced diet, excercise and sleep. Providing information about the risks of using stimulants. Adapting drug prevention methods to the context of esports.
Mental health	Providing evidence-based information on specific risk factors, symptoms and treatment option of addiction for esport players and making information on help available within games and during events. Developing measures for the early detection of problematic or addictive use patterns, and measures of intervention, for example by adapting relevant player protection practices of the gambling industry. Preparing minors to navigate age-inappropriate and otherwise potentially harmful content to minimize the adverse effects of videogame playing on their development. Initiating open conversation to raise awareness and decrease stigmatization related to excessive video gaming.
Gambling elements	Providing reliable information about the nature and risks of gambling-like elements embedded in video games. Initiating regulation of lootboxes and skin gambling, such as imposing age restrictions, and requiring gambling operator licence, and implementing harm minimization and consumer protection measures. Limiting the number of gambling commercials and team sponsorship displays during tournaments. Providing further information, preventive measures, treatment and help for those with gambling problems.
Career-path in esports	Making information available regarding possible benefits, dangers and negative consequences of pursuing an esports career. Preparing schools and educators to supporting under-age players in succeeding in their studies while pursuing a career in esports
Women in esports	Encouraging the participation of women in esports by building a support system that takes into consideration the unique challenges they face, such as the potential of encountering negative or toxic behavior.
Support of professional esports players	Providing psychological and medical help for esports players. Involving sport psychologists to help with processing failure and success, stress-management and burnout prevention. Committing to ethical gaming by education of players, and initiating open conversation on the ethical aspects, such as cheating and using other illicit methods during gaming.
Implementation	Working cooperatively and collaboratively with stakeholders (policymakers, gaming regulators, gaming industry, national and international esports organizations, healthcare and treatment providers, educational institutes, researchers, and the parents/guardians of minors). Clarifying roles, responsibilities and tasks of stakeholders. Tasking at least partially the esports industry with the responsibility to protect consumers, and providing support to health services, education, prevention and related research. Using part of the industry profits for preventive measures. Supporting collaboration with researchers to develop evidence-based problem detection and intervention systems and policies. Conducting further esports-related research to understand protective and risk factors and consequences of gaming-related problems and to support the production of effective harm prevention and intervention.

### Physical health issues

Esports training requires a high amount of training hours for an extended period of time (i.e., years), and playing videogames long-term may have several negative consequences on the lifestyle and health of players. Some of these result from the sedentary nature of videogame playing (Foti, Eaton, Lowry, & McKnight-Ely, 2011; Must & Parisi, 2009) and the lower levels of physical activity.

It is important to provide reliable information for players on the possible negative consequences on their bodies and lifestyle, such as wrist, neck, and elbow pain, weakness or numbness in the hands, carpal tunnel syndrome, skin blisters, calluses, and blood clots, as well as the risk of developing unhealthy eating habits, obesity, and sleep disorders (Chan et al., 2022). Prevention measures for such harms to players should include providing information to both the players (and in the case of minors to their guardians) regarding the importance of a balanced diet, physical exercise, and appropriate sleep to maintain a good general health condition. Education on these matters should be integral components embedded within professional esports training and providing personal counselling could also be a possible way of supporting players.

Special attention is needed to the use of stimulants. To enhance their performance, esports players may consume high amounts of these, including caffeine, energy drinks, and prescription drugs (e.g., amphetamine, methylphenidate or lisdexamfetamine) without medical supervision, which may have harmful effects (Ip et al., 2021). Medical information concerning possible dangers of using such drinks or substances, alone or in combination, should be made available for videogame players and especially for esports players. The use of illicit drugs intended to enhance the performance of gamers should also be addressed, and ideally prevented. Besides providing detailed information about the related risks of using stimulants, methods used with success in drug and gambling prevention need to be explored and adapted to the context and participants of esports.

There should be special attention to the health condition of minors, with a strong emphasis on the importance of having a healthy diet and physical exercise (Chan et al., 2022) and the esports industry should engage in corporate social responsibility (CSR) activities to promote healthier and balanced lifestyles to players (including diet, exercise, and appropriate rest) (Chan et al., 2022). The welfare-state and mass media can contribute to these campaigns to promote healthier esports consumption (Freitas, Contreras-Espinosa, & Correia, 2019).

After reviewing the current status of esports health, Schary, Jenny, and Koshy (2022) emphasized the need for health-related policies and creating specific esports-related health intervention programs, and the necessity of collaboration of healthcare and esports professionals and the involvement of the esports industry. Also, given that esports appeal to groups that are traditionally harder to influence, the potential of using esports as a vehicle to change health behavior is a possibility that needs to be further explored in collaborative work involving manufacturers, esports organizations, and researchers (Polman, Trotter, Poulus, & Borkoles, 2018).

### Mental health issues

In some cases, playing videogames can turn into an addiction (Stevens, Dorstyn, Delfabbro, & King, 2021). Although results on prevalence estimates of gaming disorder are conflicting (Lopez-Fernandez, Williams, Griffiths, & Kuss, 2019), it might impact a significant proportion of gamers. National surveys have shown higher rates in Asian countries (Liao, Chen, Huang, & Shen, 2022; Saunders et al., 2017). In the case of adult gamers, it was estimated to be 16.7% in China (Wu, Lai, Yu, Lau, & Lei, 2017), 4% in Australia (King, Herd, & Delfabbro, 2018) and 2.9% in Hungary (Király et al., 2017). More recently, Stevens et al. (2021) investigated prevalence estimates of gaming disorder comprising 53 studies (*n* = 226,247; 17 countries). The prevalence of gaming disorder was 3.05% but lower in high quality studies (1.96%). Gaming disorder prevalence rates were approximately 2.5:1 in favour of males compared to females.

It is important to provide evidence-based information on specific risk factors and symptoms (King et al., 2019), guidelines to prevent addiction symptoms (King & Delfabbro, 2017), and possibilities for therapy in case esports players and videogame players experience problems or feel they need help regarding their gaming (King et al., 2017). There is a strong need for specialist support services for mental health for gamers, who often seek the help of gambling disorder support services, that might not be perfectly prepared for the special needs and problems of gamers. Information on self-help or professional help for players needs to be made available within games (e.g., on the main menu or loading screens), and during esports events (e.g., brochures, kiosks etc). As the early detection of problematic or addictive use patterns are of special importance, relevant measures should be provided by the industry and esports clubs following the principles of screening and the need for early intervention such as giving feedback (Rumpf et al., 2018) or by the adaptation of relevant player protection practices of the gambling industry, such as introducing mandatory breaks, setting time limits, cooling-off periods, and personalized messaging (Griffiths & Pontes, 2020). If possible, digital phenotyping and sensing can serve as a reliable measure to detect problematic behavior patterns (Montag & Rumpf, 2021).

Special attention should also be given to the mental health condition of minors, including providing information and support for their family members, and protecting them from or preparing them to navigate age-inappropriate and otherwise potentially harmful content, helping them to minimize the adverse effects of videogame playing on their body image, personal development, and social relationships.

Relatedly, since stigma can be strong towards those with gaming disorder (Peter, Li, Pfund, Whelan, & Meyers, 2019), there is a chance that in some cases this can be generalized more widely to those playing videogames extensively (Galanis, Delfabbro, & King, 2021) and those engaging in videogame competitions. In a recent paper that analyzed Chinese mainstream media articles, the most frequent framing of videogames was found to be the “poison to youth” (Cao & He, 2021). Therefore, it is essential to have an open conversation (including promotional events and campaigns) concerning the potentially negative and positive effects of esports to increase awareness and decrease stigmatization.

### Gambling elements in videogames and esports

Compared to traditional sports, there is a far closer relationship between esports and gambling that not only necessitates regulation (Holden, Kaburakis, & Rodenberg, 2017) but also the protection of players. Illegal and/or unregulated gambling related to esports brings the most disrepute to related organizations from spectators (Freitas, Contreras-Espinosa, & Correia, 2021), and gambling is increasingly converging with esports in two key ways. Firstly, videogames, including popular esports titles, comprise gambling-like elements such as loot boxes, or betting with skins (i.e., in-game digital items, like visual enhancement of characters, possessing a marketplace monetary value (Greer et al., 2023), which increase the danger of developing gambling-related problems even among minors (Garea, Drummond, Sauer, Hall, & Williams, 2021; Gibson, Griffiths, Calado, & Harris, 2022; Zendle & Cairns, 2019). Reliable information about such risks should also be readily available to the esports industry, videogame players, educators, and healthcare providers. Due to the involvement of minors, parents also need to be helped to understand and manage these in-game gambling elements and the harms and risks associated with these (Király, Zhang, Demetrovics, & Browne, 2022). Moreover given the similarities that loot box buying shares with gambling (Griffiths, 2018), such activity should be regulated and access to games with loot boxes should be restricted to players who are of a legal gambling age (Zendle & Cairns, 2019). Similarly, skin gambling operators, who allow players to use skins from popular videogames for betting on esports or video-game-themed versions of casino games (Greer et al., 2019), should have to obtain a gambling operator license, impose age restrictions, and implement harm minimization and consumer protection features (Hing et al., 2021).

Betting on esports is also gaining increasing popularity (Griffiths, 2017), and esports spectatorship has been shown to have a positive association with esports gambling (Macey & Hamari, 2018), and as with any other form of gambling, esport gambling can be associated with problem gambling, problem gaming, and mental health conditions (Marchica, Richard, Mills, Ivoska, & Derevensky, 2021; Richard, Ivoska, & Derevensky, 2021). Indeed research has reported relatively high levels of problem gambling among esport bettors (Wardle, Petrovskaya, & Zendle, 2020). Moreover, the levels of problem gambling severity in some cases can even be higher among esport bettors compared to sports bettors (Gainsbury, Abarbanel, & Blaszczynski, 2017; Greer, Rockloff, Russel, & Lole , 2021). The risks of gambling on esports need to be properly addressed in policy, ensuring that esports gamblers are treated with equal (if not more care) than traditional gamblers. Existing responsible gambling tools need to be promoted and made accessible by consumers and the implementation of consumer protection measures needs to be considered, including the possibility of setting spending limits, permanent self-exclusion, and cooling-off periods without gambling (Gainsbury et al., 2017). Esports associations and event organizers should cap the number of esports gambling commercials per event in physical venues, and in event live-streams and regulate team sponsorship displays. In addition to regulation, provision of further information, preventive measures, treatment, and help for those with problems should be provided by the industry, esports associations, health care providers, and educational institutes.

### Career-path in esports

Given that a career as an esports player is becoming an option desired by many young videogame players, there should be objective information available regarding this career path, including all possible benefits as well as the actual likelihood of success, and the dangers and negative consequences that may arise (Happonen & Minashkina, 2019). Since many esports players are minors, it is essential to involve and help parents and educators understand this activity by providing them with information on esports as a profession. Schools and parents should also be prepared to support players who are still in education to be able to succeed in their studies while pursuing a potential career in esports.

### Women in esports

Esports could be a viable option for girls and women. However, as Lopez-Fernandez et al. (2019) note, although videogame playing can be beneficial for females' cognitive and social abilities, they are much less encouraged to play videogames than males. They also tend to have more negative experiences when playing videogames such as harassment from male players (McLean & Griffiths, 2019). While there has been an increase in the proportion of female players (Interactive Software Federation of Europe, 2021), the field remains male-dominated, and women are under-represented among professional esports players (Chikish, Carreras, & García, 2019). Most research is also concentrated on male gamers (Rogstad, 2021). To encourage the participation of women in esports, a support system needs to be built that not only addresses the needs of all esports participants described more generally here, but takes into consideration the unique challenges women and girls face such as the potential negative or toxic behavior women may encounter in the esports world (Imgart, 2022).

### Support of professional esports players

Similar to traditional sports, providing psychological and medical help for esports players, teams, and prospective esports players is crucial. In addition to contributing significantly to improved performance, sports psychologists can also help in processing failure and success and provide support to prevent burnout (Smith, Sharpe, Arumuham, & Birch, 2022). Since esports championships are highly competitive, stress management may also be crucial to prevent related harm (Smith, Birch, & Bright, 2019). Overall, providing psychological and medical help is indispensable in esports to support an industry growth that provides an activity that is safe from a mental health perspective. There must also be an open conversation on the ethical aspects of esports (such as cheating and using other illicit methods to enhance game performance such as the use of illegal stimulants). There must be a commitment to ethical gaming, that includes the education of players on such issues.

## Implementation

While the esports industry is expected to generate global revenue of nearly $1.38 billion (US) by the end of 2022 (Newzoo, 2022), prevention from the possible dangers that can arise from regular esports consumption for players, spectators, and gamblers must not be overlooked. To ensure optimal harm minimization, a variety of stakeholders are recommended to work in cooperation and collaboration with each other. These should include policymakers, gaming regulators, gaming industry, national and international esports organizations, healthcare and treatment providers, educational institutes, researchers, and the parents/guardians of minors. As many of the harm minimization measures listed in this paper will require input and effort from multiple stakeholders, responsibilites and tasks of all concerned should be made clear.

Policymakers should work in association with researchers and experts in the field to develop policies that address the aforementioned problems and risks, plan actions, and designate organizations and institutes on several levels that will be responsible for the implementation. This work should include the development of esports-related ethics (Peng, Dickson, Scelles, Grix, & Brannagan, 2020) to reduce cheating, match-fixing, sexism, and trolling. This should ideally be overseen by one central global governing body (Kelly, Derrington, & Star, 2022) and special attention is needed to ensure equality of access for females and minorities in esports. The role and responsibility of the esports industry needs to be defined and it should be tasked at least partially with the responsibility to protect consumers, provide information, support access to psychological and physical health services, and support any related research, as well as financially supporting education and prevention initiatives and/or programs.

In view of the enormous profits of the gaming industry and esports organizers and the potential risks described above, part of the profits should be made available for preventive measures as the gambling industry does in some jurisdictions such as the UK (Griffiths, 2009). Moreover, the gaming industry should cooperate with researchers to make use of behavioral tracking data on their players and develop machine learning algorithms to detect early signs of problem gaming, problem gambling or addictive patterns (Griffiths & Pontes, 2020). Although there are esports federations in a growing number of countries that aim to address these issues, their work needs to be supported and guided by national and international policies. Moreover, national, regional, and cultural specificities need to be addressed in relation to such issues.

Finally, further esports-related research is needed to increase the available evidence-based information in order to understand protective and risk factors for gaming-related physical and mental health issues (Wattanapisit, Wattanapisit, & Wongsiri, 2020), the nature and development of possible negative consequences of excessive play for both male and female gamers, and be able to produce more effective harm prevention and intervention (Yin et al., 2020).

## Funding sources

ZD's contribution was supported by the Hungarian National Research, Development and Innovation Office (KKP126835). OK's contribution was supported by the János Bolyai Research Scholarship of the Hungarian Academy of Sciences. HJRs contribution was supported by the German Research Foundation (DFG; FOR 2974) and the German Innovation Fund (01NVF19031).

## Authors' contribution

ZD and AC designed the project. All authors contributed to the writing of the manuscript, commented on the first draft of the manuscript, revised and approved the final version.

## Conflict of interest

ELTE Eötvös Loránd University receives funding from the Szerencsejáték Ltd. to maintain a telephone helpline service for problematic gambling. ZD has also been involved in research on responsible gambling funded by Szerencsejáték Ltd. and the Gambling Supervision Board and provided educational materials for the Szerencsejáték Ltd's responsible gambling program. The University of Gibraltar receives funding from the Gibraltar Gambling Care Foundation. PR provides training and consultancy for gambling operators as an independent consultant, he is also Chief of Safer Gambling partnerships for the Scottish charity betblocker.org which provides free gambling blocking software for people affected by gambling-related harm. Betblocker is mainly founded by UKGC LCCP RET and donations from operators in licensed jurisdictions. PR is also a Non-Executive Director for ESG Gaming not-for-profit, registered and regulated Community Interest Company. JG in her capacity as President of PRET Solutions has received funding for responsible gambling research services from gambling operators in Canada, the UK, Australia and Italy. MDG's university has received research funding from *Norsk Tipping* (the gambling operator owned by the Norwegian Government). MDG has also received funding for a number of research projects in the area of gambling education for young people, social responsibility in gambling and gambling treatment from Gamble Aware (formerly the Responsible Gambling Trust), a charitable body which funds its research program based on donations from the gambling industry. MDG regularly undertakes consultancy for various gambling companies in the area of social responsibility in gambling. However, none of the above listed funding sources are related to this article and the funding institutions/organisations had no role in the study design or the collection, analysis, and interpretation of the data, writing the manuscript, or the decision to submit the paper for publication. ZD is the chief editor of the Journal of Behavioral Addictions.
